# β-lactamase-mediated resistance in MDR-*Pseudomonas aeruginosa* from Qatar

**DOI:** 10.1186/s13756-020-00838-y

**Published:** 2020-11-01

**Authors:** Mazen A. Sid Ahmed, Faisal Ahmad Khan, Ali A. Sultan, Bo Söderquist, Emad Bashir Ibrahim, Jana Jass, Ali S. Omrani

**Affiliations:** 1 Division of Microbiology, Department of Laboratory Medicine and Pathology, Hamad Medical Corporation, P.O. Box 3050, Doha, Qatar; 2 The Life Science Centre - Biology, School of Science and Technology, Orebro University, Orebro, Sweden; 3 Department of Microbiology and Immunology, Weill Cornell Medicine-Qatar, Doha, Qatar; 4 School of Medical Sciences, Faculty of Medicine and Health, Orebro University, Orebro, Sweden; 5 Division of Infectious Diseases, Department of Medicine, Hamad Medical Corporation, Doha, Qatar; 6 Communicable Diseases Center, Hamad Medical Corporation, Doha, Qatar

**Keywords:** Beta-lactamases, MDR, *P. aeruginosa*, ST235

## Abstract

**Background:**

The distribution of β-lactam resistance genes in *P. aeruginosa* is often closely related to the distribution of certain high-risk international clones. We used whole-genome sequencing (WGS) to identify the predominant sequence types (ST) and β-lactamase genes in clinical isolates of multidrug-resistant (MDR)-*P. aeruginosa* from Qatar

**Methods:**

Microbiological identification and susceptibility tests were performed by automated BD Phoenix™ system and manual Liofilchem MIC Test Strips.

**Results:**

Among 75 MDR-*P. aeruginosa* isolates; the largest proportions of susceptibility were to ceftazidime-avibactam (n = 36, 48%), followed by ceftolozane-tazobactam (30, 40%), ceftazidime (n = 21, 28%) and aztreonam (n = 16, 21.3%). All isolates possessed Class C and/or Class D β-lactamases (n = 72, 96% each), while metallo-β-lactamases were detected in 20 (26.7%) isolates. Eight (40%) metallo-β-lactamase producers were susceptible to aztreonam and did not produce any concomitant extended-spectrum β-lactamases. High risk ST235 (n = 16, 21.3%), ST357 (n = 8, 10.7%), ST389 and ST1284 (6, 8% each) were most frequent. Nearly all ST235 isolates (15/16; 93.8%) were resistant to all tested β-lactams.

**Conclusion:**

MDR-*P. aeruginosa* isolates from Qatar are highly resistant to antipseudomonal β-lactams. High-risk STs are predominant in Qatar and their associated MDR phenotypes are a cause for considerable concern.

## Background

Due to their established efficacy and safety, anti-pseudomonal β-lactam antibiotics play a vital role in the clinical management of *P. aeruginosa* infections [1]. Key antimicrobial resistance mechanisms in *P. aeruginosa* include over-expression of efflux pumps, impermeability through porin modification or loss, target modification, and enzyme-mediated antimicrobial inactivation (e.g., β-lactamases). Multiple resistance mechanisms are frequently present in concert resulting in simultaneous resistance to multiple agents [1]. The epidemiology of β-lactamases is often closely related to the distribution of certain high-risk international clones [2]. In this study, we used whole-genome sequencing (WGS) to identify the predominant sequence types (STs) and β-lactamase genes in multi-drug resistant (MDR) *P. aeruginosa* clinical isolates from Qatar.

## Methods

The study setting, bacterial identification, antimicrobial susceptibility testing, whole genome sequencing, and statistical analysis details are provided in Additional File 1. MDR status was defined as *in-vitro* resistance to at least one agent from three or more classes of anti-pseudomonal agents [3]. β-lactamases were classified according to their molecular groups [4]. Clinical data were retrieved from the electronic healthcare system.

## Results

**Fig. 1:**
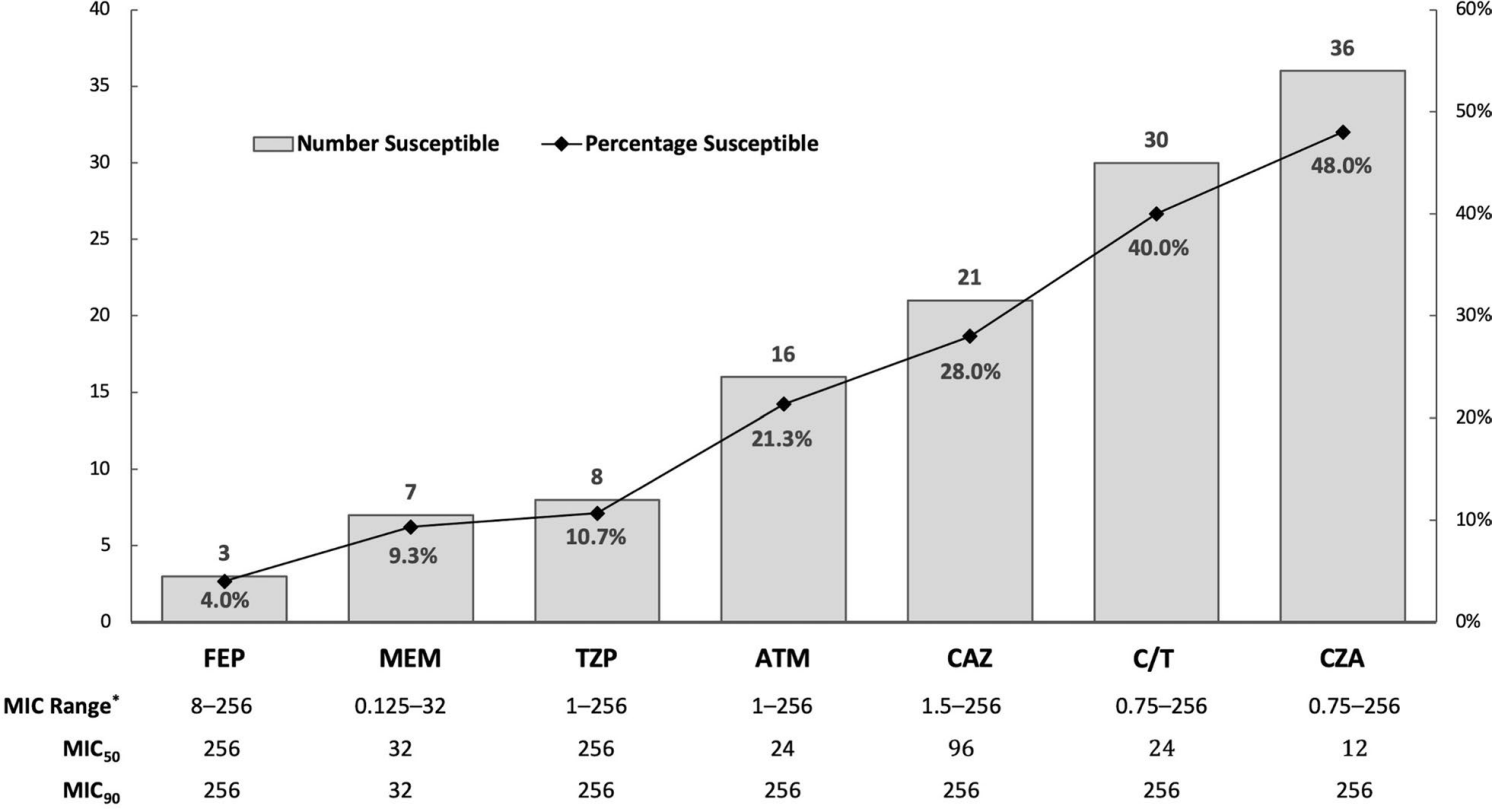
Antimicrobial susceptibility testing results for 75 MDR-*P. aeruginosa* isolates from Qatar. Columns represent number (Y axis) of isolates susceptible and line represents percentage (Z axis) of isolates susceptible to the corresponding antipseudomonal β-lactam. Reporting is based on CLSI breakpoint recommendations (M100, 30th edition—January 2020). ATM, aztreonam; CAZ, ceftazidime; CZA, ceftazidime-avibactam; C/T, ceftolozane-tazobactam; FEP, cefepime; MEM, meropenem; MIC, minimum inhibitory concentration in µg/mL, TZP, piperacillin-tazobactam

Seventy-five MDR-*P. aeruginosa* isolates were included (Additional file 1: Table S1). The largest proportions of susceptibility were to ceftazidime-avibactam [36, 48%; minimum inhibitory concentration (MIC)_50/90_ 12/256 µg/ml] and ceftolozane-tazobactam (30, 40%; MIC_50/90_ 24/256 µg/ml) (Fig. 1). Four (5.3%) isolates were resistant to all tested β-lactams except ceftazidime-avibactam, while only one (1.3%) isolate was only susceptible to ceftolozane-tazobactam (Additional file 1: Table S1).

Almost all isolates possessed Class C and Class D β-lactamases (72, 96% each). All 4 β-lactamase classes were present in 3 (4%) isolates. Metallo-β-lactamases (MBL) were detected in 20 (26.7%) isolates. Eight (40%) MBL producers were susceptible to aztreonam and did not produce any concomitant extended-spectrum β-lactamases (ESBL) (Additional file 1: Table S1).

**Fig. 2:**
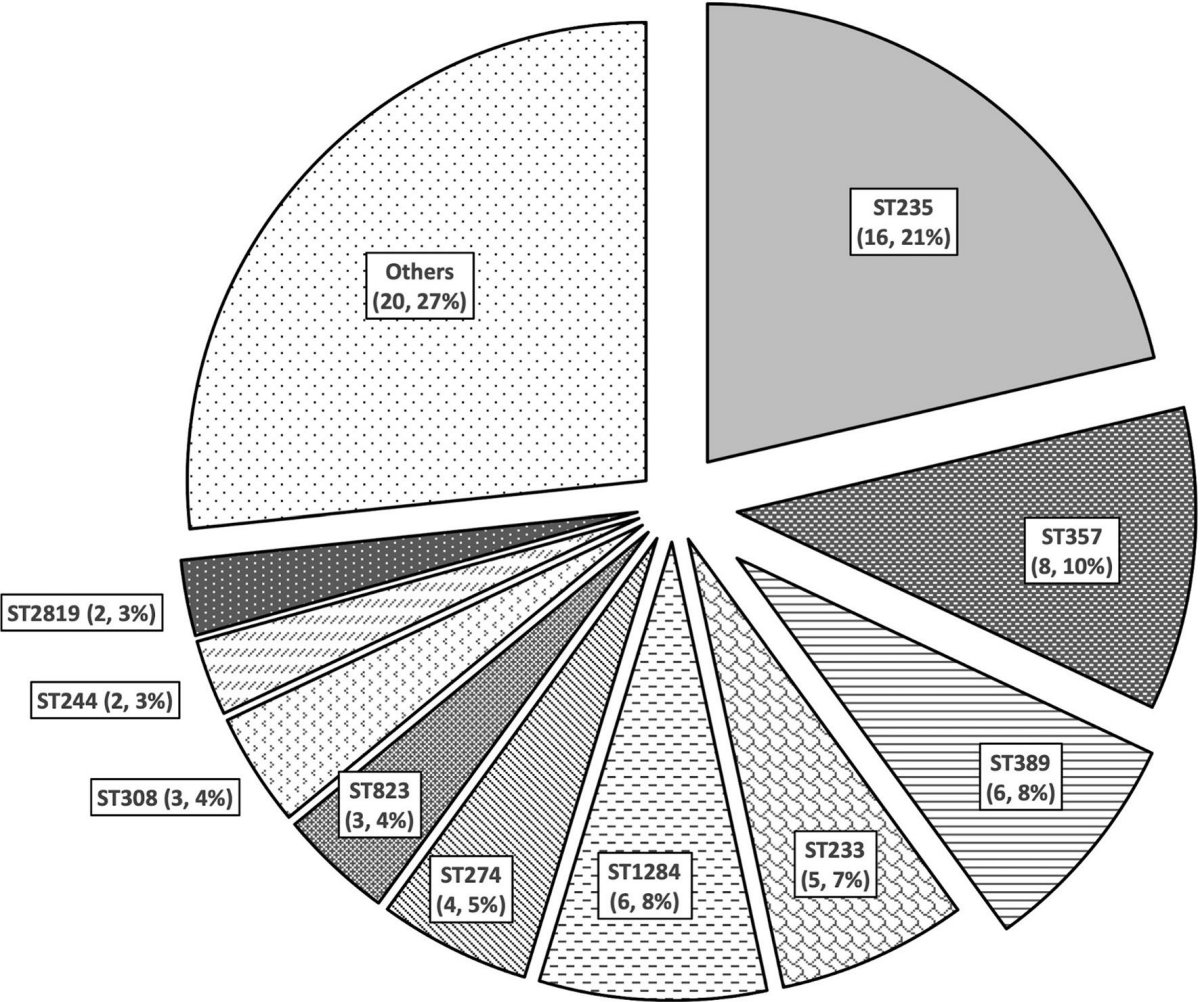
Distribution of sequence types of MDR-*P. aeruginosa* (n = 75) isolated in Qatar from 2014 to 2017

The most frequent STs identified were ST235 (16, 21.3%) and ST357 (8, 10.7%) (Fig. 2). All but one ST235 isolate were resistant to all tested β-lactam agents. Furthermore, amongst the 16 ST235 MDR-*P. aeruginosa* isolates included in this study, MBL were detected in nine (56.3%), *bla*_VEB-9_ in 8 (50%), *bla*_PDC-2_ in 15 (93.8%), and *bla*_OXA-10_ and *bla*_OXA-50_ in all 16 (100%). There were five ST233 MDR-*P. aeruginosa* isolates; all possessed *bla*_VIM-2_, *bla*_PDC-3_, *bla*_OXA-4_ and *bla*_OXA-486_, and four (80%) of them were resistant to all tested β-lactams except aztreonam. Different patterns of β-lactamase genes and β-lactam susceptibility were observed in other STs (Additional file 1: Table S2).

## Discussion

This study included data representative of the whole country, as it analyzed isolates from a national diagnostic laboratory. Notably, MDR-*P. aeruginosa* in Qatar are highly resistant to β-lactam agents. The most active β-lactam antibiotics in this study were those in combination with β-lactamase inhibitors, ceftazidime-avibactam and ceftolozane-tazobactam, were not available for clinical use at the time of the study. Yet, less than half of the isolates were susceptible. Given their recent availability for patients in Qatar, the results reported demonstrate the importance of their appropriate clinical use to minimize further loss of activity [5].

This report included 20 (26.67%) isolates that possessed 21 MBL-encoding genes (16 *bla*_VIM-2_, 2 *bla*_VIM-5,_ and 3 *bla*_IMP-2_) (Additional file 1: Tables S1 and S2). This is consistent with the known predominance of Verona integron-encoded metallo-β-lactamases (VIM), and to a lesser extent imipenemases (IMP), in *P. aeruginosa* from the Middle East [6–8]. Unlike other geographic settings, New Delhi metallo-β-lactamases (NDM) have not been detected in *P. aeruginosa* from the Arabian Peninsula [7, 9].

Apart from areas with a high prevalence of MBL in *P. aeruginosa*, the presence of Class A ESBL β-lactamases can result in resistance to ceftolozane-tazobactam [1]. Avibactam is an efficient inhibitor of Class A β-lactamases and hence ceftazidime-avibactam combination retains its activity in this situation but not ceftolozane-tazobactam [10, 11]. In a report from Spain of 24 extremely-drug resistant ST235 *P. aeruginosa* isolates, 13% were susceptible to ceftolozane-tazobactam and 58% to ceftazidime-avibactam and the predominant β-lactamases identified were VIM-2 (42%) and the Class A ESBL Guiana-Extended-Spectrum (GES)-5 (46%) [12]. Consistent with this, five out of seven ceftolozane-tazobactam-resistant, ceftazidime-avibactam-susceptible MDR-*P. aeruginosa* isolates in our study possessed class A *bla*_SHV-11_ and ESBL-encoding genes such as *bla*_VEB-9_ and *bla*_TEM-116_. Interestingly, those 7 isolates belonged to seven different STs (Additional file 1: Table S1).

The β-lactamase *bla*_VEB-9_ (19, 25.33%), formerly known as *bla*_VEB-1a_, was the most frequent ESBL gene identified in the present study [11]. *bla*_VEB-1_ is one of the most frequently reported ESBLs in *P. aeruginosa* from the Middle East including Kuwait, Saudi Arabia and Iran [13–15]. Though *bla*_VEB-9_ was reported from Thailand and Eastern Europe, to the best of our knowledge, it has not been previously reported from the Middle East [11, 16]. In this study, MDR-*P. aeruginosa* producing Vietnamese extended-spectrum beta-lactamase-9 (VEB-9) belonged to ST235 (8/16), ST357 (7/8), ST308 (1/3) and ST3022 (1/1) (Additional file 1: Tables S1 and S2). This pattern suggests dissemination within specific *P. aeruginosa* STs in Qatar that may be different from neighboring countries.

An interesting observation in this study was that 16 (21.33%) MDR-*P. aeruginosa* isolates were susceptible to aztreonam but resistant to several other antipseudomonal β-lactams tested (Additional file 1: Table S1). Aztreonam is a weak inducer of Class C enzymes and is not a substrate for Class B and narrow-spectrum Class D β-lactamases [17]. The retained aztreonam activity in these isolates despite resistance to other antipseudomonal β-lactams may be explained by the absence of Class A ESBL in those isolates. Therefore, aztreonam should be included in routine antimicrobial susceptibility testing of clinical *P. aeruginosa* isolates.

Most MDR-*P. aeruginosa* isolates included in this study belonged to five STs and had consistent β-lactamase genetic profiles and β-lactam susceptibility patterns (Additional file 1: Table S2). ST235, ST233, and ST357 are already known as high-risk clones in Qatar, Saudi Arabia, Bahrain, and the United Arab Emirates [7]. These three STs are globally disseminated MDR-*P. aeruginosa* clones [2]. Often, these strains cause regional or nationwide outbreaks, express MDR phenotypes, and are associated with high mortality [12, 18, 19]. VIM-producing ST1284 *P. aeruginosa* have been described from Brazil, and ST389 from cystic fibrosis patients in Italy [20, 21]. Both sequence types have otherwise limited geographic distribution.

## Conclusion

MDR-*P. aeruginosa* isolates from Qatar are highly resistant to antipseudomonal β-lactams. Global high-risk STs predominate in Qatar and their associated multi-resistant phenotype is a cause for considerable concern.

## Supplementary information

**Additional file 1**. Supplementary data file. 

## Data Availability

The datasets used and analyzed during the current study are available from the corresponding author on reasonable request.

## Abbreviations

CLSI: Clinical Laboratory Standards Institute
ESBL: Extended-spectrum β-lactamase
GES: Guiana-Extended-Spectrum
IMP: Imipenemase
MBL: Metallo-β-lactamase
MIC: Minimum inhibitory concentration
MDR: Multi-drug resistant
NDM: New Delhi metallo-β-lactamase
ST: Sequence type
VEB: Vietnamese extended-spectrum β-lactamase
VIM: Verona integron-encoded metallo-β-lactamase
WGS: Whole-genome sequencing
