# Supplementary material for: β-lactamase-mediated resistance in MDR-Pseudomonas aeruginosa from Qatar
Source: Antimicrob Resist Infect Control. 2020 Nov 1;9:170. doi: 10.1186/s13756-020-00838-y (PMC7603671; doi:10.1186/s13756-020-00838-y)
Supplement: Supplementary file 1 — Additional file 1. Supplementary data file. [file 13756_2020_838_MOESM1_ESM.docx]

**Additional file 1**

**β-lactamase-mediated resistance in MDR-*Pseudomonas aeruginosa* from Qatar**

Mazen A. Sid Ahmed,^1,2^ Faisal Ahmad Khan,^2^ Ali A. Sultan,^3^ Bo Söderquist,^4^ Emad Ibrahim,^1^ Jana Jass,^2^ Ali S. Omrani^5,6^

1. Department of Laboratory Medicine and Pathology, Microbiology Division, Hamad Medical Corporation, Doha, Qatar
2. The Life Science Centre - Biology, School of Science and Technology, Orebro University, Orebro, Sweden
3. Department of Microbiology and Immunology, Weill Cornell Medicine-Qatar, Doha, Qatar.
4. School of Medical Sciences, Faculty of Medicine and Health, Orebro University, Orebro, Sweden.
5. Division of Infectious Diseases, Department of Medicine, Hamad Medical Corporation, Doha, Qatar
6. Communicable Diseases Center, Hamad Medical Corporation, Doha, Qatar.

**Methods**

**Study Setting**

The study was conducted on clinical specimens received at Hamad Medical Corporation Microbiology Department during the period between October 2014 and September 2017. The department provides diagnostic microbiology services to all public hospitals in Qatar. The isolates were recovered from patients who received medical care in Hamad General Hospital (630-bedded acute care hospital), Rumailah Hospital (600 bedded long-term care hospital), and the National Center for Cancer Care and Research (46 bedded hemato-oncology center).

**Bacterial identification and antimicrobial susceptibility testing**

Bacterial identification and initial antimicrobial susceptibility testing was performed on BD Phoenix™ (Becton, Dickinson and Company, Franklin Lakes, New Jersey, United States). If required, identification was confirmed using Matrix-Assisted Laser Desorption/Ionization-Time of Flight (MALDI-TOF) mass spectrometry (Bruker Corporation, Billerica, Massachusetts). Liofilchem® MIC Test Strips (Liofilchem, Roseto degli Abruzzi, Italy) were used for minimum inhibitory concentration (MIC) determination. All commercial methods were performed according to their respective manufacturer’s instructions. *Escherichia coli* ATCC 25922, *E. coli* ATCC 35218, and *P. aeruginosa* ATCC 27853 were used as controls. Clinical Laboratory Standards Institute (CLSI) breakpoints were used for sensitive and resistant classification [1].

**Whole genome sequencing**

Out of 525 MDR-*P. aeruginosa* isolates which were collected between October 2014 and September 2017, 75 isolates were subsequently processed for WGS performed by Eurofins Genomics using Illumina HiSeq 2000 system (Illumina, San Diego, California).

The clean reads were assembled using SPAdes, Version 3.13.0 (Center for Algorithmic Biotechnology, St Petersburg, Russia) [2]. Multi-locus sequence type (MLST) of MDR-*P. aeruginosa* isolates was performed using MLST 1.8 (Center for Genomic Epidemiology), based on seven housekeeping genes [3]. Genes associated with antimicrobial resistance were predicted using the Comprehensive Antibiotic Resistance Database (CARD), Version 1.2.0 (McMaster University, Hamilton, Ontario) [4].

**Statistical analysis**

The study data are presented as frequency and percentage. Statistical analyses were conducted using Microsoft Excel (Microsoft Corporation, Redmond, Washington).

**Table S1.** Demographic characteristics and molecular characterization and susceptibility testing results for 75 MDR-*P. aeruginosa* isolates.

| **Isolate** | **ST** | **Collection m-y** | **Hospital** | **Location** | **Sample type** | **Molecular β-lactamase Class Detected in the Isolate** | | | | **Antimicrobial susceptibility test** | | | | | | |
| --- | --- | --- | --- | --- | --- | --- | --- | --- | --- | --- | --- | --- | --- | --- | --- | --- |
|  |  |  |  |  |  | **Class A** | **Class B** | **Class C** | **Class D** | **FEP** | **MEM** | **TZP** | **ATM** | **CAZ** | **CZA** | **C/T** |
| PA1 | ST235 | Sep-14 | HGH | OPD | SST |  | IMP-1 | PDC-2 | OXA-488 | R | R | R | R | R | R | R |
| PA9 | ST235 | Oct-14 | HGH | IPF | Urine |  | VIM-2 | PDC-2 | OXA-488 | R | R | R | R | R | R | R |
| PA26 | ST235 | Nov-14 | HGH | ICU | SST |  | VIM-2 | PDC-2 | OXA-17 OXA-488 | R | R | R | R | R | R | R |
| PA27 | ST235 | Nov-14 | HGH | OPD | SST |  | IMP-1 | PDC-2 | OXA-488 | R | R | R | R | R | R | R |
| PA37 | ST235 | Dec-14 | HGH | IPF | Other |  | VIM-2 | PDC-2 | OXA-17 OXA-488 | R | R | R | R | R | R | R |
| PA99 | ST235 | Mar-15 | HGH | OPD | Urine | VEB-9 |  | PDC-2 | OXA-10 OXA-488 | R | R | R | R | R | R | R |
| PA128 | ST235 | Apr-15 | HH | ICU | RT |  | VIM-2 | PDC-2 | OXA-17 OXA-114 OXA-488 | R | R | R | R | R | R | R |
| PA129 | ST235 | Apr-15 | HGH | OPD | Urine |  | VIM-2 | PDC-2 | OXA-114 OXA-488 | R | R | R | R | R | R | R |
| PA131 | ST235 | May-15 | HGH | OPD | Urine | VEB-9 |  | PDC-2 | OXA-10 OXA-488 | R | R | R | R | R | R | R |
| PA134 | ST235 | May-15 | HGH | OPD | Urine | VEB-9 |  | PDC-2 | OXA-10 OXA-488 | R | R | R | R | R | R | R |
| PA143 | ST235 | May-15 | HGH | IPF | Urine | VEB-9 |  | PDC-2 | OXA-10 OXA-488 | R | R | R | R | R | R | R |
| PA169 | ST235 | Jun-15 | HGH | OPD | Urine | VEB-9 |  | PDC-2 | OXA-10 OXA-488 | R | R | R | R | R | R | R |
| PA176 | ST235 | Jul-15 | HGH | IPF | SST |  | VIM-2 | PDC-2 | OXA-488 | R | R | R | R | R | R | R |
| PA203 | ST235 | Sep-15 | HGH | OPD | RT | VEB-9 |  |  | OXA-10 OXA-114 OXA-488 | R | S | S | R | S | S | R |
| PA209 | ST235 | Sep-15 | HGH | OPD | Urine | VEB-9 |  | PDC-2 | OXA-10 OXA-488 | R | R | R | R | R | R | R |
| PA250 | ST235 | Dec-15 | HGH | IPF | Urine | VEB-9 |  | PDC-2 | OXA-488 | R | R | R | R | R | R | R |
| PA16 | ST357 | Oct-14 | HGH | IPF | RT | VEB-9 |  | PDC-3 | OXA-10 OXA-50 | R | R | R | R | R | R | R |
| PA17 | ST357 | Oct-14 | HGH | OPD | Urine | VEB-9 |  | PDC-3 | OXA-10 OXA-50 | R | R | R | R | R | R | R |
| PA40 | ST357 | Dec-14 | HGH | IPF | SST | VEB-9 |  | PDC-3 | OXA-10 OXA-50 | R | R | R | R | R | R | R |
| PA41 | ST357 | Dec-14 | HGH | IPF | Urine | TEM-126 VEB-9 |  | PDC-3 | OXA-10 OXA-50 | R | R | R | R | R | R | R |
| PA130 | ST357 | May-15 | HGH | IPF | SST | VEB-9 |  | PDC-3 | OXA-10 OXA-50 | R | R | R | R | R | R | R |
| RPA135 | ST357 | May-15 | HGH | OPD | Urine |  |  | PDC-3 | OXA-50 | R | S | R | R | S | S | R |
| PA208 | ST357 | Sep-15 | HGH | ICU | Blood | CTX-M15 VEB-9 SHV-11 | VIM-5 | PDC-3 | OXA-10 OXA-50 | R | R | R | R | R | R | R |
| PA212 | ST357 | Oct-15 | HGH | IPF | Blood | VEB-9 | VIM-5 | PDC-3 | OXA-10 OXA-50 | R | R | R | R | R | R | R |
| PA19 | ST389 | Oct-14 | HGH | OPD | RT |  |  | PDC-1 | OXA-50 | R | R | S | R | S | S | S |
| PA140 | ST389 | May-15 | HGH | OPD | RT | TEM-116 |  | PDC-1 | OXA-50 | R | R | R | R | R | R | R |
| PA175 | ST389 | Jul-15 | HGH | OPD | RT | TEM-116 |  | PDC-1 | OXA-50 | R | R | R | R | R | R | R |
| PA253 | ST389 | Dec-15 | HGH | OPD | RT |  |  | PDC-1 | OXA-50 | R | R | R | R | R | R | R |
| PA311 | ST389 | Mar-16 | HGH | OPD | RT |  |  | PDC-1 | OXA-50 | R | R | R | R | R | R | R |
| PA350 | ST389 | Jun-16 | HGH | IPF | RT |  |  | PDC-1 | OXA-50 | R | R | R | R | R | R | R |
| PA6 | ST1284 | Sep-14 | HGH | ICU | SST |  |  | PDC-3 | OXA-488 | S | R | R | R | R | S | S |
| PA20 | ST1284 | Oct-14 | HGH | OPD | Urine | TEM-116 |  | PDC-3 | OXA-488 | R | R | R | R | S | S | S |
| PA148 | ST1284 | May-15 | HGH | IPF | Blood |  |  | PDC-3 | OXA-488 | R | R | R | S | S | S | S |
| PA166 | ST1284 | Jun-15 | HGH | IPF | SST |  |  | PDC-3 | OXA-488 | R | R | R | R | S | S | S |
| PA187 | ST1284 | Aug-15 | RH | IPF | SST |  |  | PDC-3 | OXA-488 | R | R | R | R | S | S | S |
| PA196 | ST1284 | Aug-15 | HGH | OPD | Urine | CTX-M15 SHV-11 |  |  | OXA-488 | R | R | R | R | R | S | S |
| PA51 | ST233 | Dec-14 | HGH | ICU | SST |  | VIM-2 | PDC-3 | OXA-4 OXA-486 | R | R | R | S | R | R | R |
| PA199 | ST233 | Sep-15 | RH | IPF | RT |  | VIM-2 | PDC-3 | OXA-4 OXA-486 | R | R | R | S | R | R | R |
| PA220 | ST233 | Oct-15 | HGH | IPF | Blood |  | VIM-2 | PDC-3 | OXA-4 OXA-114 OXA-486 | R | R | R | R | R | R | R |
| PA508 | ST233 | Jul-17 | HGH | IPF | Blood |  | VIM-2 | PDC-3 | OXA-4 OXA-486 | R | R | R | S | R | R | R |
| PA527 | ST233 | Aug-17 | HGH | IPF | Blood |  | VIM-2 | PDC-3 | OXA-4 OXA-486 | R | R | R | S | R | R | R |
| PA5 | ST274 | Oct-14 | HGH | ICU | RT | TEM-116 |  | PDC-10 | OXA-486 | R | R | R | R | S | S | S |
| PA32 | ST274 | Nov-14 | HGH | ICU | RT |  |  | PDC-10 | OXA-486 | R | R | R | R | S | S | S |
| PA110 | ST274 | Mar-15 | HGH | IPF | RT | TEM-116 |  | PDC-10 | OXA-486 | R | R | R | R | S | S | S |
| NS PA161 | ST274 | Jun-15 | HGH | ICU | RT |  |  | PDC-10 | OXA-486 | R | R | R | R | S | S | S |
| PA36 | ST244 | Dec-14 | HGH | OPD | SST | TEM-1 | VIM-2 IMP-1 | PDC-1 | OXA-4 OXA-10 OXA-129 OXA-50 LCR-1 | R | R | R | R | R | R | R |
| NS NS NS NS PA126 | ST244 | Apr-15 | HGH | ICU | SST |  |  | PDC-1 | OXA-10 OXA-50 | R | R | R | R | S | S | S |
| PA11 | ST308 | Oct-14 | HGH | IPF | RT | VEB-9 |  | PDC-7 | OXA-488 | R | R | R | R | R | R | R |
| PA12 | ST308 | Oct-14 | HGH | OPD | Urine | VEB-9 |  | PDC-7 | OXA-488 | R | R | R | R | R | R | R |
| PA98 | ST308 | Mar-15 | HGH | OPD | Urine | VEB-9 |  | PDC-7 | OXA-488 | R | R | R | R | R | S | R |
| PA125 | ST823 | Apr-15 | HGH | IPF | RT |  | VIM-2 | PDC-7 |  | R | R | R | S | R | R | R |
| PA183 | ST823 | Jul-15 | NCCCR | IPF | Blood |  | VIM-2 | PDC-7 |  | R | R | R | S | R | R | R |
| PA200 | ST823 | Sep-15 | HGH | IPF | RT | SHV-11 | VIM-2 | PDC-7 |  | R | R | R | S | R | R | R |
| PA207 | ST2819 | Sep-15 | HGH | ICU | RT |  |  | PDC-5 | OXA-50 | R | R | R | R | R | S | S |
| PA232 | ST2819 | Nov-15 | HGH | IPF | Blood |  |  | PDC-5 | OXA-50 | R | R | R | S | R | S | S |
| RRPA241 | ST1076 | Dec-15 | HGH | IPF | Blood | CTX-M15 |  | PDC-7 | OXA-50 | R | R | R | R | S | S | S |
| PA323 | ST446 | Apr-16 | HGH | IPF | RT |  |  | PDC-7 | OXA-50 | R | R | R | R | S | S | S |
| PA142 | ST560 | May-15 | HGH | IPF | SST |  |  | PDC-10 | OXA-488 | R | R | R | R | R | S | S |
| PA447 | ST598 | Feb-17 | RH | IPF | Blood |  |  | PDC-3 | OXA-50 | R | R | R | R | R | S | S |
| PA180 | ST639 | Jul-15 | HGH | ICU | RT |  |  | PDC-3 | OXA-486 | R | R | R | S | S | S | S |
| PA66 | ST664 | Jan-15 | HGH | IPF | SST |  |  | PDC-8 | OXA-4 OXA-10 OXA-50 | R | R | R | R | R | R | R |
| PA420 | ST699 | Dec-16 | RH | IPF | Blood |  |  | PDC-1 | OXA-50 | R | R | R | R | R | R | S |
| PA498 | ST773 | Jun-17 | HGH | IPF | Blood |  | VIM-2 | PDC-7 | OXA-50 | R | R | R | S | R | R | R |
| PA10 | ST381 | Oct-14 | HGH | IPF | RT |  |  | PDC-3 | OXA-50 | S | R | R | R | S | S | S |
| RRRRPA4 | ST17 | Sep-14 | HGH | OPD | SST |  |  | PDC-8 | OXA-50 | R | S | R | R | S | S | S |
| PA154 | ST27 | Jun-15 | HGH | ICU | RT | TEM-116 |  | PDC-5 | OXA-50 | R | R | R | R | R | S | R |
| PA428 | ST179 | Dec-16 | HGH | IPF | SST |  |  | PDC-8 | OXA-14 OXA-50 | R | R | R | R | R | R | S |
| PA86 | ST252 | Feb-15 | HGH | ICU | RT |  |  | PDC-3 | OXA-486 | R | R | R | R | R | S | S |
| PA190 | ST253 | Sep-15 | HGH | IPF | SST |  |  | PDC-9 | OXA-488 | R | R | R | S | S | S | S |
| PA123 | ST292 | Apr-15 | HGH | IPF | Blood | CARB-3 |  | PDC-5 | OXA-50 | R | R | R | S | R | S | S |
| PA349 | ST310 | Jun-16 | HGH | IPF | RT |  |  | PDC-3 | OXA-488 | R | R | R | S | S | S | S |
| PA78 | ST313 | Jan-15 | HGH | OPD | RT |  |  | PDC-7 | OXA-488 | S | R | S | S | S | S | S |
| PA263 | ST348 | Jan-16 | RH | IPF | Blood |  |  | PDC-5 | OXA-50 | R | R | R | R | R | S | S |
| PA119 | ST3022 | Apr-15 | HGH | ICU | RT | VEB-9 |  |  | OXA-10 OXA-50 | R | R | R | R | R | S | R |
| PA457 | ST3043 | Mar-17 | HGH | IPF | Blood |  |  |  | OXA-50 | R | S | S | R | S | S | S |

Susceptibility was reported according to Clinical Laboratory Standards Institute (CLSI) breakpoints (17)

m, month; y, year; HGH, Hamad General Hospital; RH, Rumailah Hospital; NCCCR, National Center for Cancer Care and Research; ICU, intensive care unit; IPF, inpatient floors; OPD, outpatient department; RT, respiratory tract; SST, skin and soft tissue; FEP, cefepime; MEM, meropenem; TZP, piperacillin-tazobactam; ATM, aztreonam; CAZ, ceftazidime; CZA, ceftazidime-avibactam; C/T, ceftolozane-tazobactam. R; resistance, S; susceptible

**Table S2.** Most frequent sequence types and associated β-lactamase genes and susceptibility to β-lactam antipseudomonal agents.

| **Sequence Type** | **β-lactamase Genes Detected** | **Number (%) susceptible** | | | | | | |
| --- | --- | --- | --- | --- | --- | --- | --- | --- |
|  |  | **FEP** | **MEM** | **TZP** | **ATM** | **CAZ** | **CZA** | **C/T** |
| ST235 (n = 16) | *bla*_VEB-9_ (8, 50%)  *bla*_VIM-2_ (6, 38%)  *bla*_IMP-1_ (2, 13%)  *bla*_PDC-2_ (15, 94%)  *bla*_OXA-10_ (16, 100%)  *bla*_OXA-488_ (16, 100%) | 0 | 1  (6%) | 1  (6%) | 0 | 1  (6%) | 1  (6%) | 0 |
| ST357 (n = 8) | *bla*_VEB-9_ (7, 88%)  *bla*_VIM-5_ (2, 25%)  *bla*_PDC-3_ (8, 100%)  *bla*_OXA-10_ (8, 100%)  *bla*_OXA-50_ (8, 100%) | 0 | 1  (13%) | 0 | 0 | 1  (13%) | 1  (13%) | 0 |
| ST389 (n = 6) | *bla*_TEM-116_ (2, 33%)  *bla*_PDC-1_ (6, 100%)  *bla*_OXA-50_ (6, 100%) | 0 | 0 | 1  (17%) | 0 | 1  (17%) | 5  (83%) | 3  (50%) |
| ST1284 (n = 6) | *bla*_TEM-116_ (1, 17%)  *bla*_CTX-M15_ (1, 17%)  *bla*_PDC-3_ (5, 83%)  *bla*_OXA-488_ (6, 100%) | 1 | 0 | 0 | 1  (17%) | 4  (67%) | 6  (100%) | 5  (83%) |
| ST233 (n = 5) | *bla*_VIM-2_ (5, 100%)  *bla*_PDC-3_ (5, 100%)  *bla*_OXA-4_ (5, 100%)  *bla*_OXA-486_ (5, 100%) | 0 | 0 | 0 | 4  (80%) | 0 | 0 | 0 |
| ST274 (n = 4) | *bla*_TEM-116_ (2, 50%)  *bla*_PDC-10_ (4, 100%)  *bla*_OXA-486_ (4, 100%) | 0 | 0 | 0 | 3  (75%) | 2  (50%) | 2  (50%) | 1  (25%) |
| ST823 (n = 3) | *bla*_VIM-2_ (3, 100%)  *bla*_PDC-7_ (3, 100%) | 0 | 0 | 0 | 3  (100%) | 2  (67%) | 2  (67%) | 1  (33%) |
| ST308 (n = 3) | *bla*_VEB-9_ (3, 100%)  *bla*_PDC-7_ (3, 100%) | 0 | 2  (67%) | 2  (67%) | 0 | 0 | 1  (33%) | 0 |
| ST244 (n = 2) | *bla*_TEM-1_ (1, 50%)  *bla*_VIM-2_ (1, 50%)  *bla*_IMP-1_ (1, 50%)  *bla*_PDC-1_ (2, 100%)  *bla*_OXA-4_ (1, 50%)  *bla*_OXA-10_ (2, 100%)  *bla*_OXA-50_ (2, 100%)  *bla*_OXA-129_ (1, 50%)  *bla*_LCR-1_ (1, 50%) | 0 | 0 | 0 | 0 | 1  (50%) | 1  (50%) | 1  (50%) |
| ST2819 (n = 2) | *bla*_PDC-5_ (2, 100%)  *bla*_OXA-50_ (2, 100%) | 0 | 0 | 0 | 1  (50%) | 0 | 2  (100%) | 2  (100%) |

ATM, aztreonam; CAZ, ceftazidime; CZA, ceftazidime-avibactam; C/T, ceftolozane-tazobactam; FEP, cefepime; MEM, meropenem; TZP, piperacillin-tazobactam

**References**

1. Clinical Laboratory Standards Institute. Performance Standards for Antimicrobial Susceptibility Testing: CLSI Supplement M100, 30th edition Wayne, PA, USA: CLSI; 2020 [Available from: http://em100.edaptivedocs.net/GetDoc.aspx?doc=CLSI%20M100%20ED29:2019&scope=user.

2. Bankevich A, Nurk S, Antipov D, Gurevich AA, Dvorkin M, Kulikov AS, et al. SPAdes: a new genome assembly algorithm and its applications to single-cell sequencing. J Comput Biol. 2012;19(5):455-77.

3. Larsen MV, Cosentino S, Rasmussen S, Friis C, Hasman H, Marvig RL, et al. Multilocus sequence typing of total-genome-sequenced bacteria. J Clin Microbiol. 2012;50(4):1355-61.

4. Jia B, Raphenya AR, Alcock B, Waglechner N, Guo P, Tsang KK, et al. CARD 2017: expansion and model-centric curation of the comprehensive antibiotic resistance database. Nucleic Acids Res. 2017;45(D1):D566-d73.
